# Associations Between HIV-Related Stigma, Trust, and Testing Behaviors Among the General U.S. Adult Population

**DOI:** 10.1007/s10461-025-04684-0

**Published:** 2025-03-24

**Authors:** Sue Hyon Kim, Stephen Bonett, José Bauermeister, Alison M. Buttenheim, Laura E. Starbird

**Affiliations:** 1 University of Pennsylvania School of Nursing, 418 Curie Boulevard, Philadelphia, PA 19104 USA; 2 Center for Health Incentives and Behavioral Economics, University of Pennsylvania, Philadelphia, PA USA; 3 Leonard Davis Institute of Health Economics, University of Pennsylvania, Philadelphia, PA USA

**Keywords:** HIV stigma, Social stigma, HIV testing, Trust, United States

## Abstract

HIV testing is essential to achieving the 95-95-95 targets, yet lifetime HIV testing rates in the United States fall below established guidelines. Building on similar healthcare contexts where stigma hinders access and trust mitigates its negative effect, this exploratory study examined the relationship between HIV-related stigma (HRS) and testing behavior, focusing on the role of trust in healthcare providers (HCPs). We used data from the 2022 General Social Survey, a nationally representative sample of the general population. HRS was evaluated across three domains: perceived social discrimination towards people living with HIV (PLWH), avoidance due to unfounded fear of transmission, and moral judgement. Weighted stratified logistic regression was employed to examine how the relationship between HRS and testing behavior varied across different levels of trust in HCPs. Among participants with low trust in HCPs, the avoidance of PLWH due to unfounded fears was associated with lower odds of undergoing HIV testing. In the high trust group, none of the HRS domains were associated with HIV testing; instead, testing was linked to the individual’s engagement in HIV-risk behaviors. Our findings reveal a complex interplay between HRS, trust, and testing behavior, highlighting the need for collective action to address misconceptions about HIV transmission and promote awareness of risk behaviors, with concurrent efforts to foster trust in HCPs.

## Background

The significance of early HIV diagnosis for treatment and viral suppression is underscored by the new 95-95-95 targets, aiming for 95% of all people living with HIV (PLWH) to know their HIV status by 2030 [1]. Recognizing HIV testing as the crucial first step towards achieving these targets, the Centers for Disease Control and Prevention (CDC) guidelines for the United States (U.S.) recommend that everyone between the ages of 13 and 64 undergo HIV testing at least once as part of routine healthcare [2]. Although there has been promising progress with increases in testing rates, less than half of U.S. adults have ever been tested for HIV [3]. Additionally, cisgender women and heterosexual individuals who each account for nearly 20% of new HIV diagnoses remain under-studied in the literature regarding their HIV testing behavior [4].

Along with the global 95-95-95 HIV targets, efforts have also been made to reduce stigma and discrimination associated with HIV, which some consider a “fourth target” [5]. Despite reported declines of stigma towards PLWH, HIV remains a highly stigmatized health condition, subject to misconceptions and resulting in suboptimal prevention and treatment outcomes [6]. The HIV Stigma Framework delineates internalized, anticipated, and enacted stigma mechanisms among PLWH [7, 8], which also operates on structural levels that lead to discrimination and inequalities in institutional practices and daily life [9]. Various forms of HIV-related stigma perpetuate negative social norms and public attitudes that are often endorsed by the general population [10]. Such stigma frequently manifests through social disapproval, avoidance, and moral judgements, and has been identified as a barrier to HIV testing, impeding the attainment of the 95-95-95 targets [10, 11].

Several interpersonal support strategies have been shown to counter the undesirable effects of stigma. Healthcare satisfaction and social support were able to moderate the negative impact of HIV-related stigma on medication adherence and psychological distress [12, 13]. Trust in one’s neighbor has shown promise in promoting timely HIV testing among Black men who have sex with men (MSM), possibly through the effects of social cohesion and social capital [14]. The importance of a trusting relationship with healthcare providers (HCPs) is evident in other areas where stigma acts as a barrier to accessing care, such as in mental health services [15]. Distrust in HCPs is often rooted in concerns about confidentiality and social norms, which ultimately discourage individuals from seeking help [16]. Similar dynamics have been observed across other healthcare settings, including cancer screening examinations and vaccinations, where trust in the healthcare system and in physicians increased the utilization of preventative health services [17, 18]. Thus, trust in HCPs may mitigate the deterring effects of HIV-related stigma, encouraging individuals with higher trust to engage in preventative health behaviors, such as HIV testing, despite any stigma they may personally hold.

Research exploring the potential impact of trust in HCPs on HIV-related stigma and testing behavior remains relatively scarce among the general population. Therefore, our study examined the relationship between HIV testing behavior and HIV-related stigma in a nationally representative sample of U.S. adults. Moreover, to develop a greater understanding of the factors influencing HIV testing in the context of stigma and trust, we also examined whether the association between HIV testing and HIV-related stigma varied depending on participants’ trust in HCPs.

## Methods

### Data and Sample

We analyzed data from the General Social Survey (GSS), a cross-sectional descriptive survey assessing sociopolitical attitudes and behaviors among a nationally representative sample of the U.S. population. This survey includes adults aged 18 years or older who reside in non-institutionalized housing and speak either English or Spanish. Data collection took place from May to December 2022 using a multi-stage cluster sampling strategy and multi-mode approach including face-to-face interviews, telephone interviews, or web surveys. Additional completed responses were obtained by oversampling individuals from Black, Hispanic, and Asian demographics. Further details regarding the GSS can be found elsewhere [19]. Ethical review and approval were waived by the University of Pennsylvania Institutional Review Board for the utilization of publicly available and fully de-identified data.

In this secondary analysis, we included participants who provided responses related to HIV testing history, HIV-related stigma, and trust in HCPs.

### Measures

#### HIV Testing Status

The GSS included survey items that asked participants whether they had ever undergone HIV testing (including blood test or oral swab) and their experiences. We dichotomized responses into ‘no’ versus ‘yes’ regarding participants’ history of getting tested for HIV. In line with the study’s aim to explore associations between stigma, trust, and the intentional behavior of HIV testing, respondents who were unaware of their HIV testing history and selected ‘Do not know/Cannot choose’ were classified as not having undergone HIV testing.

#### Stigmatization Related To HIV

HIV-related stigma was assessed across three domains: the perceived social discrimination towards PLWH, individual actions marked by avoidance of PLWH due to unfounded fear of transmission, and personal moral judgement against PLWH. These items were adopted from tools developed by Zelaya et al. [20] and the Kaiser Family Foundation [21]. Participants expressed their level of agreement or disagreement with the following statements using a 4-point Likert scale: (a) There is a lot of discrimination against people with HIV in this country today, (b) I would be afraid to be around a person with HIV because I would be worried I could get infected, and (c) People who have HIV have participated in immoral activities. Higher scores indicated a stronger agreement with the statements.

#### Trust in Healthcare Providers

Trust in HCPs was assessed using participants’ responses to a question from the GSS regarding trust in doctors. Participants conveyed their level of agreement or disagreement with the statement ‘All things considered, doctors can be trusted’ on a 5-point Likert scale, ranging from 1 (Strongly agree) to 5 (Strongly disagree). To facilitate analysis, these responses were reverse-coded, so that higher scores reflected higher levels of trust. Reverse-coded responses were subsequently stratified into two trust levels to explore potential group differences: low trust (responses 1, 2, and 3) and high trust (responses 4 and 5).

#### Frequently Cited HIV-Associated Risk Factors

Commonly recognized risk factors for HIV, as identified in prior literature and CDC guidelines, were utilized [22, 23]. These included the number of sexual partners in the past 12 months, condom use during recent sexual encounters, engagement in transactional sex, injection drug use, and binge drinking. Participants were considered to have engaged in HIV-associated risk behavior if they had two or more sexual partners in the past 12 months [24, 25], engaged in unprotected or transactional sex, practiced injection drug use [22, 23] or reported binge drinking (consuming more than 4 drinks of alcohol per day [26]) on multiple occasions each month [22, 27]. Subsequently, an HIV risk behavior index that ranged from zero to five was calculated by aggregating participants’ responses to these risk factors, with each manifestation contributing one point to the index.

MSM are also recognized as having a higher risk of acquiring HIV. To maintain a consistent maximum score, this characteristic was not included into the HIV risk behavior index but analyzed as a separate variable. Data on participants’ gender and the gender of their sexual partners in the last 12 months were cross-tabulated to identify MSM or those with partners of both genders. This information was coded as a binary variable distinguishing MSM and non-MSM participants.

#### Sociodemographic Characteristics

Sociodemographic characteristics included as covariates in our analyses were age (in years), gender (man, woman, transgender or gender nonconforming), race (White, Black, Other), educational degree (less than high school, high school, associate/junior college, bachelor’s, graduate), and employment status (employed full time, work part time, seeking employment, voluntary unemployment).

### Data Analysis

Participant characteristics and responses to HIV testing experiences, HIV-related stigma, and trust were summarized using descriptive statistics. Missing data were not differential with respect to HIV testing behavior, stigma, or trust. We employed logistic regression models to identify stigma domains and factors associated with HIV testing behavior, stratifying participants into two groups based on their level of trust in HCPs (low trust vs. high trust). However, low variability in HIV testing behaviors among MSM individuals and transgender or gender nonconforming participants led to quasi-complete separation, resulting in unreliable coefficients and wide confidence intervals. Recognizing the distinct experiences of HIV testing among this group [28], we focused our analyses on HIV testing behaviors in the non-MSM and cisgender population. All analyses used post-stratification weights provided by the GSS to address potential sampling and nonresponse bias and were conducted using R Statistical Software (v4.4.1; R Core Team, 2023).

## Results

### Participant Characteristics and Responses To Main Variables

Table 1 displays participant characteristics and responses in both the weighted and unweighted samples. All results discussed will be based on the weighted analyses. The mean age of the sample of non-MSM and cisgender participants included in the analyses was 47.8 (SD = 17.2) years. More than half identified as woman (*n* = 448; 55.1%) and as White (*n* = 632.1; 78.9%). Just over 10% of participants reported having an education level below high school (*n* = 93; 11.4%). The majority of participants were either employed full-time or voluntarily unemployed, such as being retired, in school, keeping house, or in another situation where they were not seeking work (*n* = 705.3; 86.8%). While most participants reported having one or no sex partners in the past 12 months (*n* = 734.2; 91%), those who reported having sex indicated low levels of condom use in their previous sexual encounters (*n* = 126.5; 16.4%). Only 5.3% of participants reported engaging in transactional sex (*n* = 42.2), while fewer than 5% engaged in injection drug use and daily binge drinking.

**Table 1:** Participant characteristics and responses

Characteristics	Unweighted sample (*N* = 834)	Weighted sample			
		Total sample (*N* = 829^†^)	Analyzed sample (*N* = 813^†^)	Unanalyzed sample (*N* = 16^†, ‡^)	*p*-value
Age	49.6 ± 17.7 years	47.6 ± 17.2 years	47.8 ± 17.2 years	38.3 ± 16 years	**0.02** ^*^
Gender (*n* = 831)					**< 0.001** ^**^
Man	370 (44.5%)	375.9 (45.3%)	365.3 (44.9%)	10.5 (66.7%)	
Woman	453 (54.5%)	448.0 (54.0%)	448.0 (55.1%)	-	
Trans & nonconforming	8 (1.0%)	5.3 (0.6%)	-	5.3 (33.3%)	
Race (*n* = 825)					**0.00** ^*^
White	607 (73.6%)	639.6 (78.0%)	632.1 (78.9%)	7.5 (41.2%)	
Black	116 (14.1%)	88.3 (10.8%)	83.5 (10.4%)	4.8 (26.3%)	
Other	102 (12.3%)	91.5 (11.2%)	85.6 (10.7%)	5.9 (32.5%)	
Education					0.27
< High school	70 (8.4%)	93.5 (11.2%)	93.0 (11.4%)	0.5 (2.5%)	
High school	381 (45.7%)	396.1 (47.6%)	387.3 (47.6%)	8.8 (45.8%)	
≥ College	383 (45.9%)	342.9 (41.2%)	333.0 (41.0%)	9.9 (51.7%)	
Employment status (*n* = 832)					0.08
Full time	389 (46.8%)	401.0 (48.2%)	388.3 (47.8%)	12.7 (66.5%)	
Part time	79 (9.5%)	76.7 (9.2%)	72.9 (9.0%)	3.8 (19.9%)	
Seeking employment	41 (4.9%)	34.8 (4.2%)	34.6 (4.2%)	0.3 (1.4%)	
Voluntary unemployment^§^	323 (38.8%)	319.3 (38.4%)	317.0 (39.0%)	2.3 (12.2%)	
Sex partners in past year (*n* = 830)					**< 0.001** ^**^
0 or 1	731 (88.1%)	746.8 (90.5%)	734.2 (91.0%)	12.6 (65.6%)	
2–4	80 (9.6%)	63.1 (7.6%)	57.9 (7.2%)	5.2 (27.2%)	
5 or more	19 (2.3%)	15.7 (1.9%)	14.4 (1.8%)	1.4 (7.2%)	
No condom use (*n*=785)	638 (81.3%)	656.3 (83.3%)	643.0 (83.6%)	13.2 (72.6%)	0.19
Transactional sex (*n*=816)	52 (6.4%)	44.0 (5.4%)	42.2 (5.3%)	1.8 (9.8%)	0.36
Injection drug use (*n*=831)	25 (3.0%)	22.1 (2.7%)	21.5 (2.7%)	0.6 (3.1%)	0.86
Alcohol ≥ 4 drinks/day (*n*=830)					0.52
Never	517 (62.3%)	490.3 (59.0%)	476.7 (58.7%)	13.6 (71.2%)	
Once a month or less often	196 (23.6%)	200.0 (24.1%)	198.9 (24.5%)	1.1 (5.9%)	
Several times a month	89 (10.7%)	86.35 (10.4%)	83.0 (10.2%)	3.3 (17.4%)	
Several times a week	22 (2.7%)	39.2 (4.7%)	38.1 (4.7%)	1.1 (5.5%)	
Daily	6 (0.7%)	15.2 (1.8%)	15.2 (1.9%)	-	
HIV risk behavior index (*n*=767)	1.17 ± 0.68	1.18 ± 0.63	1.18 ± 0.62	1.47 ± 0.86	0.16
Main variables					
Ever received HIV testing	303 (36.3%)	260.0 (31.2%)	246.8 (30.3%)	13.3 (69.2%)	**0.00** ^*^
Perceived social discrimination	2.86 ± 0.89	2.81 ± 0.89	2.79 ± 0.89	3.46 ± 0.77	**<0.001** ^**^
Unfounded avoidance	1.78 ± 0.98	1.85 ± 1.02	1.86 ± 1.02	1.48 ± 0.87	**0.04** ^*^
Moral judgement	1.69 ± 0.91	1.68 ± 0.88	1.69 ± 0.88	1.35 ± 0.62	**0.03** ^*^
Trust in HCP	3.53 ± 0.94	3.55 ± 0.93	3.55 ± 0.93	3.48 ± 0.83	0.72

*p < .05, **p < .001, ^†^Numbers may not add up due to rounding ^‡^Men who have sex with men and transgender or gender nonconforming individuals ^§^Retired, in school, keeping house, or other

*HCP* healthcare provider; *Moral judgement* moral judgements against people with HIV; *Perceived social discrimination* perceived level of discrimination towards people with HIV; *Unfounded avoidance* fear of being near people with HIV due to unfounded concerns about transmission.

**Fig. 1:**
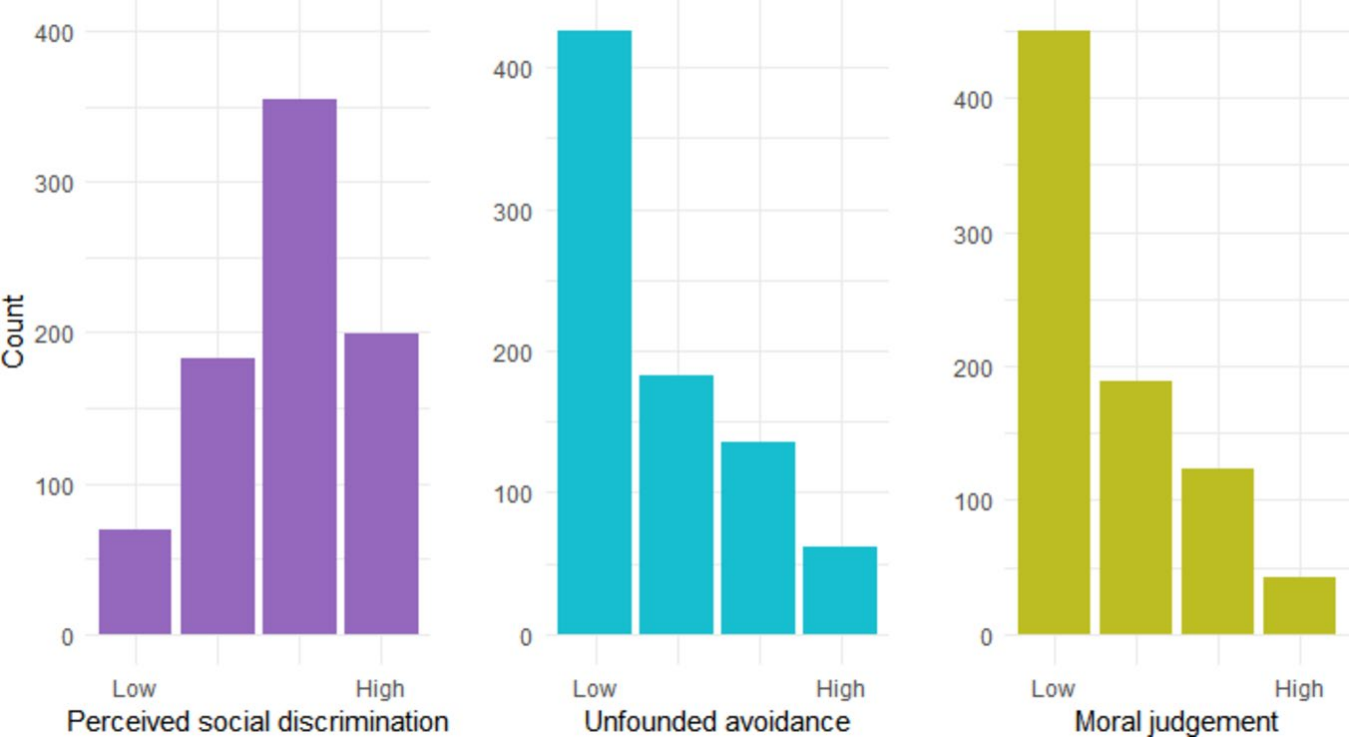
Distribution of perceived public stigma and personal stigma

Approximately one-third of the participants had ever undergone HIV testing (*n* = 246.8; 30.3%). The mean score for the perceived social discrimination towards PLWH was 2.79 (SD = 0.89) out of a possible 4. The mean scores for avoidance of PLWH due to unfounded fear of transmission and moral judgement against PLWH were 1.86 (SD = 1.02) and 1.69 (SD = 0.88), respectively, with responses skewed to the right. A discrepancy was noted between the distribution of perceived social discrimination and the two specific attitudes towards PLWH, indicating that respondents perceived a more negative social climate surrounding HIV than reflected in their personal actions or beliefs (Fig. 1). The trust in HCPs measure had a mean score of 3.55 (SD = 0.93) out of a possible 5.

Statistically significant demographic differences were observed in age, gender, and race when compared to the MSM and transgender or nonconforming counterparts who were excluded from the analyses. The excluded MSM and transgender or nonconforming individuals reported having more sex partners in the past year and demonstrated significantly different responses in terms of HIV-related stigma. There were no notable differences in other HIV-associated risk behaviors or in the overall HIV risk behavior index. The excluded sample had more than twice the percentage of HIV-tested participants (30.3% vs. 69.2%), which explained the quasi-complete separation observed in our initial analysis.

### Factors Associated with HIV Testing Behavior across Trust Levels

None of the three domains of HIV-related stigma were associated with HIV testing behavior in the full sample of non-MSM and cisgender participants (Table 2). The odds of being tested increased by 1.60 times for every additional HIV-associated risk behavior (95%CI = 1.077–2.374). A significant sociodemographic difference in HIV testing behavior was observed with regard to age (OR = 0.969, 95% CI = 0.952–0.987).

**Fig. 2:**
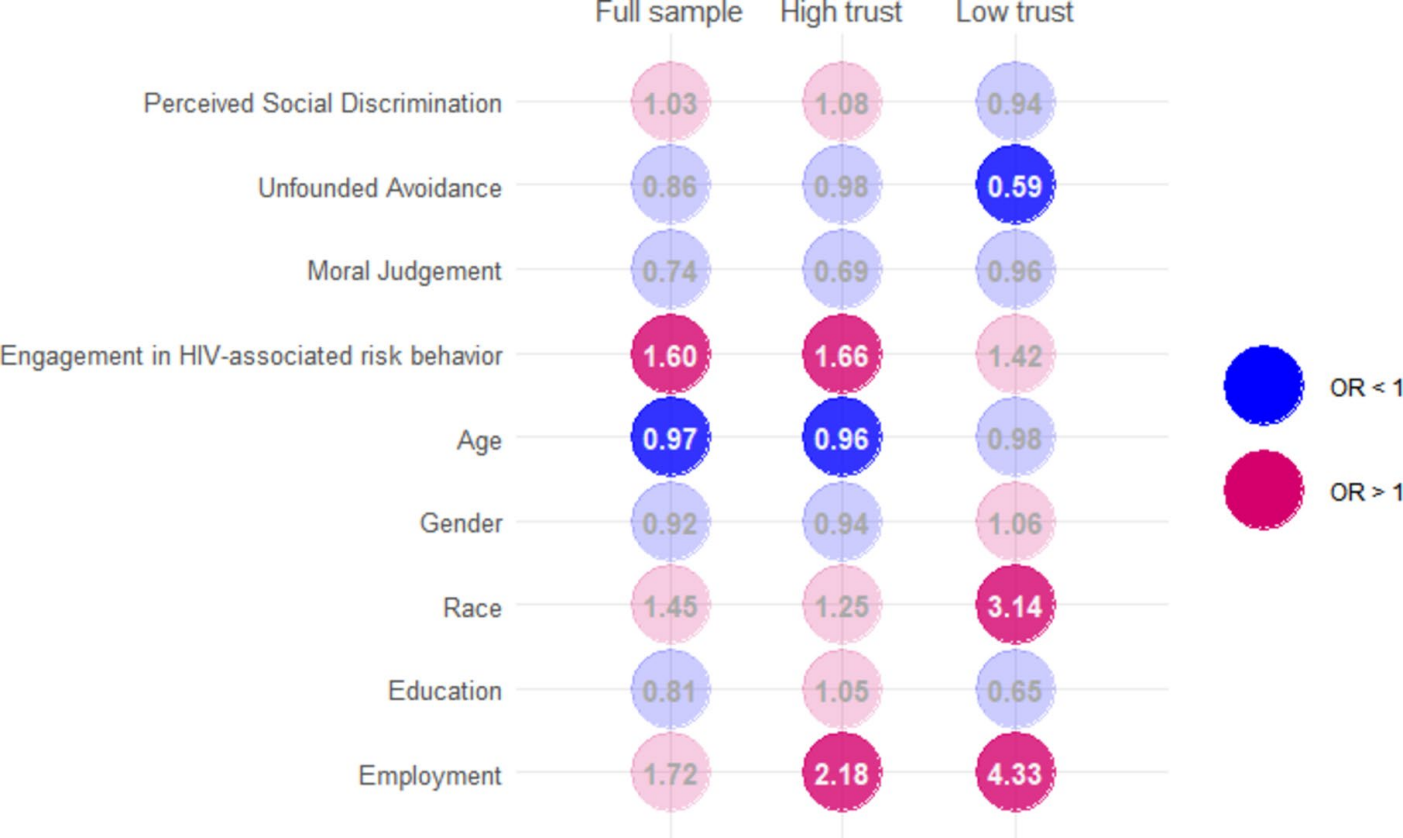
Differences in HIV testing behavior across trust levels. Results from weighted stratified logistic regression. Red indicates odds ratios (ORs) greater than 1, while blue indicates ORs less than 1. Mean ORs weighted by p-values were used as a summary measure for predictors with multiple categories. When no individual ORs were statistically significant (i.e., *p* >.05), the unweighted mean OR was calculated across categories as a summary estimate. Color intensity reflects statistical significance, with non-significant values displayed in faded colors and significant values shown in stronger colors

When examining the relationship between HIV-related stigma and testing behaviors across different levels of trust in HCPs, we found that stigma was not associated with the odds of undergoing HIV testing among participants with high trust in HCPs. However, higher levels of avoidance of PLWH due to unfounded fear of transmission were associated with reduced odds of undergoing HIV testing among participants with low trust, even after controlling for covariates (OR = 0.594, 95% CI = 0.366–0.966). Additionally, engagement in HIV-associated risk behaviors was linked to increased odds of undergoing HIV testing among participants with higher trust in HCPs (OR = 1.658, 95% CI = 1.004–2.737), whereas this positive association was not observed among those with low trust (Fig. 2).

**Table 2:** Factors associated with HIV testing behavior across trust levels (weighted results)

Predictors	Full analyzed sample (*N* = 813)		High trust in HCP (*N* = 486)		Low trust in HCP (*N* = 327)	
	OR (95% CI)	*p*-value	OR (95% CI)	*p*-value	OR (95% CI)	*p*-value
Stigma						
Perceived social discrimination	1.034 (0.749–1.428)	0.837	1.083 (0.740–1.585)	0.681	0.939 (0.574–1.537)	0.803
Unfounded avoidance	0.856 (0.621–1.182)	0.345	0.983 (0.697–1.387)	0.924	0.594 (0.366–0.966)	.036^*^
Moral judgement	0.744 (0.513–1.078)	0.118	0.695 (0.462–1.045)	0.080	0.961 (0.554–1.669)	0.888
Potential risk factors						
HIV risk index	1.599 (1.077–2.374)	**0.020** ^*^	1.658 (1.004–2.737)	**0.048** ^*^	1.415 (0.876–2.285)	0.155
Sociodemographics						
Age	0.969 (0.952–0.987)	**< 0.001** ^**^	0.960 (0.942–0.979)	**< 0.001** ^**^	0.980 (0.955–1.005)	0.121
Gender ^a^						
Woman	0.925 (0.525–1.628)	0.786	0.935 (0.484–1.808)	0.842	1.062 (0.433–2.607)	0.895
Race ^b^						
Black	2.093 (0.930–4.709)	0.074	1.529 (0.548–4.263)	0.417	3.143 (1.133–8.719)	**0.028** ^*^
Other	0.801 (0.355–1.808)	0.593	0.973 (0.365–2.596)	0.957	0.428 (0.144–1.270)	0.126
Education ^c^						
< Highschool	0.702 (0.250–1.973)	. 502	0.972 (0.303–3.114)	0.962	0.594 (0.154–2.295)	0.449
Highschool	0.920 (0.554–1.529)	0.748	1.119 (0.586–2.136)	0.733	0.706 (0.316–1.577)	0.394
Employment status ^d^						
Part time	2.092 (0.814–5.377)	0.125	1.160 (0.300-4.492)	0.830	4.331 (1.132–16.573)	**0.032** ^*^
Seeking employment	1.920 (0.524–7.034)	0.324	2.001 (0.454–8.819)	0.359	1.402 (0.221–8.896)	0.719
Voluntary unemployment	1.480 (0.792–2.766)	0.219	2.181 (1.065–4.468)	**0.033** ^*^	0.571 (0.210–1.555)	0.272
Likelihood ratio chi-squared	88.099 [13]	< 0.001^**^	65.939 [13]	< 0.001^**^	55.591 [13]	< 0.001^**^

*p < .05, **p < .001; *Reference group.*^a^ Man, ^b^ White, ^c^ ≥College, ^d^ Full time employment; Moral judgement = moral judgements against people with HIV; Perceived social discrimination = perceived level of discrimination towards people with HIV; Unfounded avoidance = fear of being near people with HIV due to unfounded concerns about transmission

Among the covariates, older age was associated with lower odds of HIV testing in the high trust group (OR = 0.960, 95% CI = 0.942–0.979) and Black individuals were more likely to undergo testing in the low trust group (OR = 3.143, 95% CI = 1.133–8.719). Various forms of employment status were significant factors in the likelihood of HIV testing across the strata.

## Discussion

In this study, we investigated the association between HIV testing behaviors and HIV-related stigma among the general population, exploring whether this relationship varied based on trust in HCPs. Slightly more than 30% had undergone HIV testing, which is consistent with previous reports on HIV testing rates that still fall below CDC guidelines [2, 3, 29].

Most participants acknowledged the presence of discrimination against PLWH; however, the levels of personal attitudes towards PLWH, such as unfounded avoidance and moral judgements, were moderately low. Our findings align with prior longitudinal data from the Netherlands, where more than a third of the PLWH reported stigmatizing experiences, despite reductions in stigma within their close social networks, such as family and friends [30]. This suggests that while some social environments have become more supportive, stigmatization remains a persistent issue in other areas of life for PLWH. Furthermore, this may reflect misperceived norms within the general population regarding the magnitude of HIV-related stigma. Although our study found no direct association between perceived social discrimination and the likelihood of HIV testing, addressing these misperceptions remains important, as they could discourage individuals from undergoing HIV testing if they expect more stigma than actually exists [31].

Unfounded fear of HIV transmission led to the avoidance of PLWH and was identified as a deterrent to seeking HIV testing among our non-MSM and cisgender sample with lower trust in HCPs. This underscores the negative impact of misinformation on HIV transmission, highlighting the importance of rectifying common myths about HIV, as discussed in previous literature [32]. This negative relationship did not hold true for participants in the high trust group, which is consistent with prior studies demonstrating the positive influence of trust in HCPs and credible government information sources on accurate knowledge, healthcare utilization, and adherence [33–35]. It can be inferred that individuals with lower trust in HCPs may be more susceptible to misinformation or fear regarding HIV transmission, as they lack confidence in their provider’s capacity to offer accurate information. On the other hand, those with higher trust are more likely to turn to HCPs for reliable information, thereby mitigating the negative impact of this form of stigma on their HIV testing behaviors.

An interesting trend in our study, although not statistically significant, is the greater likelihood of HIV testing among individuals in the high trust group who perceived greater social discrimination towards PLWH. This pattern was not observed in the low trust group. This apparent protective effect in the high trust group is unexpected, as it seemingly contradicts previous literature endorsing HIV-related stigma as a barrier to testing [11]. However, it is important to note that this domain of HIV-related stigma measured in our study pertains to the perception of the social climate around PLWH, rather than internalized or anticipated stigma. In some cases, such as in our sample of non-MSM and cisgender individuals who are less likely to identify with HIV-related stigma, perceived societal discrimination against a stigmatized group may have a protective effect- especially when coupled with their trust and confidence in the healthcare system’s ability to provide unbiased care regardless of HIV testing outcomes or societal attitudes.

Varying levels of trust in HCPs led to different patterns in HIV testing, with engagement in HIV-associated risk behaviors serving as a stronger motivator for those with greater trust in their providers. While perceptions of HIV risk may not always align with actual HIV risk [36], our finding of increased awareness and self-help behavior is promising. Unfortunately, among individuals with low trust in HCPs, HIV testing likelihood was not influenced by involvement in HIV-associated risk behaviors. This may be due to a lack of awareness of HIV-associated risk factors within the low trust group, along with concerns about confidentiality and fear of mistreatment if diagnosed with HIV during testing. Hence, public initiatives focused on raising awareness of HIV-associated risk behaviors should also aim to enhance trust in HCPs and the healthcare system to achieve greater participation in HIV testing. Moreover, individuals with lower trust in HCPs often exhibit decreased healthcare utilization and may avoid seeking HIV testing in conventional healthcare settings [37]. Therefore, expanding the use of self-test kits could be an effective strategy to encourage testing among those who recognize the need for it but are hesitant to seek testing in a clinical setting, provided that simultaneous efforts are made to ensure the reporting of positive results that link to adequate HIV care [38].

Our analysis identified a trend where younger and Black individuals were more likely to undergo HIV testing, reflecting the demographics of PLWH in the U.S [39]. Participant age and race were significant factors associated with HIV testing behavior in the stratified regression models, with age significantly related to testing behavior in the high trust group and race in the low trust group. This could be attributed to generational factors, with older individuals possibly showing stronger resistance to HIV testing due to influences from a past era of intense HIV stigmatization and a perceived lower risk of HIV, which trust in HCPs alone may not overcome [40, 41]. On the other hand, trust in HCPs attenuated the racial differences in HIV testing among our sample. Black communities bear a disproportionately high burden of HIV, and social initiatives focus on raising awareness of this issue within these populations [39]. As a result, Black individuals may recognize a higher personal or community-level risk that prompts proactive testing behaviors, especially when trust in HCPs is lower. In contrast, those with higher trust may base their decision to undergo HIV testing on other individual-level factors, such as engagement in HIV-associated risk behaviors, leading us back to our previous discussion on fostering trust to leverage self-help behaviors. Different forms of employment status were also significant across the stratified regression models. Individuals with part-time jobs or voluntary unemployment status had higher odds of undergoing HIV testing, which may be related to their increased free time and flexibility to engage with the healthcare system [42]. However, the broad categories of the sociodemographic variables used in this study warrant further research to gain a more nuanced understanding of how sociodemographic factors relate to HIV testing behavior and trust in HCPs.

This study has several limitations. First, the exclusion of MSM and transgender or gender nonconforming individuals limits the generalizability of our findings to these groups. Additionally, the cross-sectional design prevents us from inferring causality. It does not account for potential changes in participants’ views of HIV-related stigma that may have differed between the time of HIV testing and the time of responding to the survey. Furthermore, the GSS did not include data on the HIV status of the participants, which could potentially act as a confounding factor impacting perceptions of HIV-related stigma and testing behavior. However, our study makes a unique contribution by including cisgender women and heterosexual individuals who are under-represented in the literature. These groups account for a considerable portion of new HIV diagnoses in the U.S. and would greatly benefit from increased testing [4]. Our findings offer a more comprehensive understanding of HIV testing behavior, stigma, and the potential role of trust in HCPs within a nationally representative sample of the general population.

## Conclusion

Individuals from the general population are rarely the focus of public initiatives or HIV research [43, 44]. However, given the stagnant progress in the HIV care continuum among these individuals in certain regions [45], there is a pressing need to uncover the dynamics driving their behaviors related to HIV testing, prevention, and management. We examined the interplay between HIV-related stigma and trust in HCPs that influenced HIV testing behavior among non-MSM and cisgender samples from the general population. Our findings highlight the need for collective actions to address prevalent misconceptions about HIV transmission and increase awareness of risk behaviors, while fostering trust in HCPs. Our study significantly contributes to the existing body of knowledge on HIV perception and behavior, informing future studies and interventions aimed at improving HIV care for all populations.

## Data Availability

The data that support the findings of this study are openly available at the GSS Data Explorer website at gssdataexplorer.norc.org.
